# Comprehensive left atrial flow component analysis reveals abnormal flow patterns in paroxysmal atrial fibrillation

**DOI:** 10.1152/ajpheart.00614.2023

**Published:** 2023-12-22

**Authors:** Sophia Bäck, Jonas Lantz, Iulia Skoda, Lilian Henriksson, Anders Persson, Lars O. Karlsson, Carl-Johan Carlhäll, Tino Ebbers

**Affiliations:** ^1^Unit of Cardiovascular Sciences, Department of Health, Medicine and Caring Sciences, Linköping University, Linköping, Sweden; ^2^Center for Medical Image Science and Visualization, Linköping University, Linköping, Sweden; ^3^Department of Cardiology in Linköping, Linköping University, Linköping, Sweden; ^4^Department of Health, Medicine and Caring Sciences, Linköping University, Linköping, Sweden; ^5^Department of Radiology, Medicine and Caring Sciences, Linköping University, Linköping, Sweden; ^6^Department of Clinical Physiology in Linköping, Medicine and Caring Sciences, Linköping University, Linköping, Sweden

**Keywords:** atrial fibrillation, flow component analysis, hemodynamics, left atrium

## Abstract

Left atrial (LA) blood flow plays an important role in diseases such as atrial fibrillation (AF) and atrial cardiomyopathy since alterations in the blood flow might lead to thrombus formation and stroke. Using traditional techniques, such as echocardiography, atrial flow velocities can be measured at the pulmonary veins and the mitral valve, but a comprehensive understanding of the three-dimensional atrial flow field is missing. Previously, ventricular flow has been analyzed using flow component analysis, revealing new insights into ventricular flow and function. Thus, the aim of this project was to develop a comprehensive flow component analysis method for the LA and explore its utility in 21 patients with paroxysmal atrial fibrillation compared with a control group of 8 participants. The flow field was derived from time-resolved CT acquired during sinus rhythm using computational fluid dynamics. Flow components were computed from particle tracking. We identified six atrial flow components: conduit, reservoir, delayed ejection, retained inflow, residual volume, and pulmonary vein backflow. It was shown that conduit flow, defined as blood entering and leaving the LA within the same diastolic phase, exists in most subjects. Although the volume of conduit and reservoir is similar in patients with paroxysmal AF in sinus rhythm and controls, the volume of the other components is increased in paroxysmal AF. Comprehensive quantification of LA flow using flow component analysis makes atrial blood flow quantifiable, thus facilitating investigation of mechanisms underlying atrial dysfunction and can increase understanding of atrial blood flow in disease progression and stroke risk.

**NEW & NOTEWORTHY** We developed a new comprehensive approach to atrial blood component analysis that includes both conduit flow and residual volume and compared the flow components of atrial fibrillation (AF) patients in sinus rhythm with controls. Conduit and reservoir flow were similar between the groups, whereas components with longer residence time in the left atrium were increased in the AF group. This could add to the pathophysiological understanding of atrial diseases and possibly clinical management.

## INTRODUCTION

A key function of the left atrium (LA) is to act as a reservoir for blood coming from the lungs and to lead it efficiently to the ventricle (1). However, in some patients, the atrial function and blood flow are impaired, such as in atrial fibrillation (AF) or atrial cardiomyopathy (2). These disruptions lead to an increased risk of atrial thrombus formation and stroke (3), especially in aging populations. Better understanding of how these diseases alter the atrial blood flow could provide insights both in the disease mechanism and potentially identify patients with a higher risk of stroke.

Over the cardiac cycle, three distinct atrial phases have been identified: reservoir, conduit, and booster pump (4). The reservoir phase is during ventricular systole, when atrial volume increases because it is collecting blood coming from the lungs. During the following conduit phase, the mitral valve (MV) opens and blood flows from the atrium to the ventricle. During the early ventricular filling, due to ventricular relaxation, the atrial volume decreases passively while the atrium continues to receive blood from the lungs. The booster pump occurs later in ventricular filling when the atrium contracts actively, which increases the atrial pressure and decreases the blood flow from the lungs. In some individuals, atrial blood is not only pushed into the left ventricle (LV) but also retrograde toward the pulmonary veins (PV) (4, 5). The phase of ventricular systole corresponds to atrial diastole and vice versa. To avoid confusion, in the following text, we use the ventricular phase as a reference, e.g., diastole refers to ventricular diastole and systole refers to ventricular systole.

Commonly, atrial function is evaluated using 2- or 3-dimensional (2-D or 3-D) echocardiography, by analyzing the atrial volume change (6). However, this method does not assess the actual flow patterns. Cardiac blood flow can also be analyzed using 4-D flow MRI or by imaging-based computational fluid dynamics (CFD) simulations. In 4-D flow MRI, blood velocities are measured directly; however, the spatial resolution is in the range of 2–3 mm, which results in only a few voxels covering the left atrial appendage (LAA) (7). When simulating the blood flow based on time-resolved CT images, the intracardiac flow patterns are comparable to 4-D flow MRI, with higher spatial resolution (8, 9).

One way of analyzing cardiac blood flow in a more detailed manner is flow component analysis (10, 11). With this method, the blood flow in the left and right ventricle was separated into four flow components, based on particle traces. This method defines direct flow as the volume of blood that enters and leaves the ventricle within one cardiac cycle. Retained inflow describes the blood volume entering the ventricle but not leaving. Delayed ejection is blood that leaves the ventricle after being there since at least the previous cardiac cycle. The residual volume describes blood that neither enters nor leaves the ventricle during a cardiac cycle. Flow component analysis of the left ventricle (LV) has increased our understanding of LV flow and has been shown to predict functional capacity (12), as well as it showed alterations in the ventricular blood flow in patients with paroxysmal atrial fibrillation (13) and after cardioversion (14).

Particle-based component analysis has also been applied to the left atrium. Dillon-Murphy et al. (15) used a very similar approach to the ventricular method in two patients and applied it to atrial flow simulations with CFD. They seeded the atrial volume with particles right before the mitral valve opens, when the atrium is at its largest volume. Then, they used the same four components as used by Eriksson et al. (11) for the atrial flow analysis and tracked them forward over ventricular diastole and backward over ventricular systole. With this technique, blood entering the atrium when the mitral valve is closed and leaving in the following diastole can be identified. However, it does not track blood that travels through the atrium as a conduit component, entering and leaving during diastole. Gaeta et al. (16) also tracked particles in the left atrium in three subjects, but with a different approach, analyzing 4-D flow MRI data. They released particles in the pulmonary veins continuously over one cardiac cycle, starting at the beginning of systole. They define direct flow as the volume of particles that leaves the atrium in the tracked cardiac cycle. Furthermore, they identify retained inflow as blood entering but not leaving the atrium and reversed flow as particles that enter the atrium and leave through the pulmonary veins. With this approach, the direct flow also contains the conduit flow, but it does not identify other components, such as the residual volume.

So far, the atrial conduit and reservoir flow have only been described based on echocardiography measurements of the velocity at the pulmonary veins and mitral valve, but not through actual tracking of the blood flow. To broaden our understanding of these components and the overall atrial blood flow, the aim of this project was to develop an approach to analyze the atrial blood flow components, including both conduit flow and residual volume, and apply it to patients with paroxysmal atrial fibrillation and a control group to identify differences in these groups that could be linked to an increased risk of stasis in the AF group.

## MATERIALS AND METHODS

### Patient Information

This study includes 21 patients with paroxysmal AF and 8 control subjects. All participants were recruited at Linköping University Hospital. Details on the exclusion criteria can be found in Ref. (17). The paroxysmal AF group received a time-resolved CT before catheter ablation, whereas the control group was referred for a clinical coronary CT angiography.

The study has been approved by the Local Ethics Board (Regionala etikprövningsnämnden i Linköping, 2018/275-31 and 2015/396-31). All study participants provided informed written consent for the study.

### CT Acquisition and Flow Simulation

To calculate the blood flow, CFD simulations based on time-resolved CT were performed. All participants received a time-resolved CT acquired with a dual-source CT scanner (SOMATOM Force, Siemens Healthineers, Erlangen, Germany). All participants received iodinated contrast medium. The images were reconstructed to 288–476 slices with 512 × 512 pixels and 20 timeframes, each representing 5% of the RR interval.

The CFD simulations were conducted using Ansys Fluent version 2019 R3. Patient-specific cardiac wall motion was prescribed in the models based on the time-resolved CT acquisitions (9). The simulations included both the left atrium and the left ventricle. At the pulmonary veins and the aorta, a constant pressure boundary condition was prescribed, and the flow was driven by patient-specific atrial and ventricular wall motion. Thus, the total amount of blood flowing through the pulmonary veins and mitral valve is defined by the cardiac motion. The constant pressure boundary condition leads to the cross-sectional area of the PV defining the specific flow rate. The computational meshes had an element edge length range of 0.25–2 mm, with smaller elements at the wall. The meshes contained between 5 and 12 million cells, depending on the size of the heart and the cardiac phase, which was found to be sufficient in a mesh-sensitivity study. The temporal resolution was 5e-4 s, blood viscosity was set to 3.5e-3 Pa s, and density was set to 1,060 kg/m^3^. Stagnant flow in the atrium was estimated through the calculation of the blood residence time, as described previously. The details of the CT acquisition and CFD simulations can be found in Ref. (17).

### Flow Component Analysis

The main idea of the flow component analysis for the left atrium was to combine particles seeded from the end-systolic atrial volume (volume seeding) with particles seeded continuously from the pulmonary veins over a full cardiac cycle (pulmonary vein seeding). The atrial volume was seeded with particles on an isotropic Cartesian grid with a spacing of 2 mm, such that each particle represents a volume of 0.008 mL. The particle trajectories were computed in ParaView version 5.11 using the particle path filter that integrates the velocity field with the 4th-order Runge–Kutta method and a constant step size. They were tracked both forward and backward in time for a complete cardiac cycle.

For the particle tracking from the pulmonary veins, a plane was manually defined at the interface between the atrial cavity and each pulmonary vein. The distance between the seeds at the pulmonary veins was 1 mm, and new particles were released every 0.025 s. Sensitivity analysis of the temporal and spatial resolutions can be found in the Appendix. The volume of each particle was calculated according to *Eq. 1*:

(*1*) $$V_{\text{particle}}=A\times v_{\text{norm}}\times \text{d}t$$

where *A* is the area represented by the particle on the seed plane (1 mm^2^), *v*_norm_ is its velocity component normal to the plane of the pulmonary vein, and d*t* is the time step. Thus, the total sum of the volumes of the entering particles is the same as the amount of blood entering the LA during the cardiac cycle. The seeding distances for both approaches were found to be sufficiently small after comparing several different seeding distances.

After the particle trajectories were computed, their position over time was analyzed in MATLAB Version R2022b. Since the simulated data included both the LA and LV, moving masks were generated that indicate the geometry of the LA in each time step to be able to identify when a particle left the LA.

**Figure 1.**
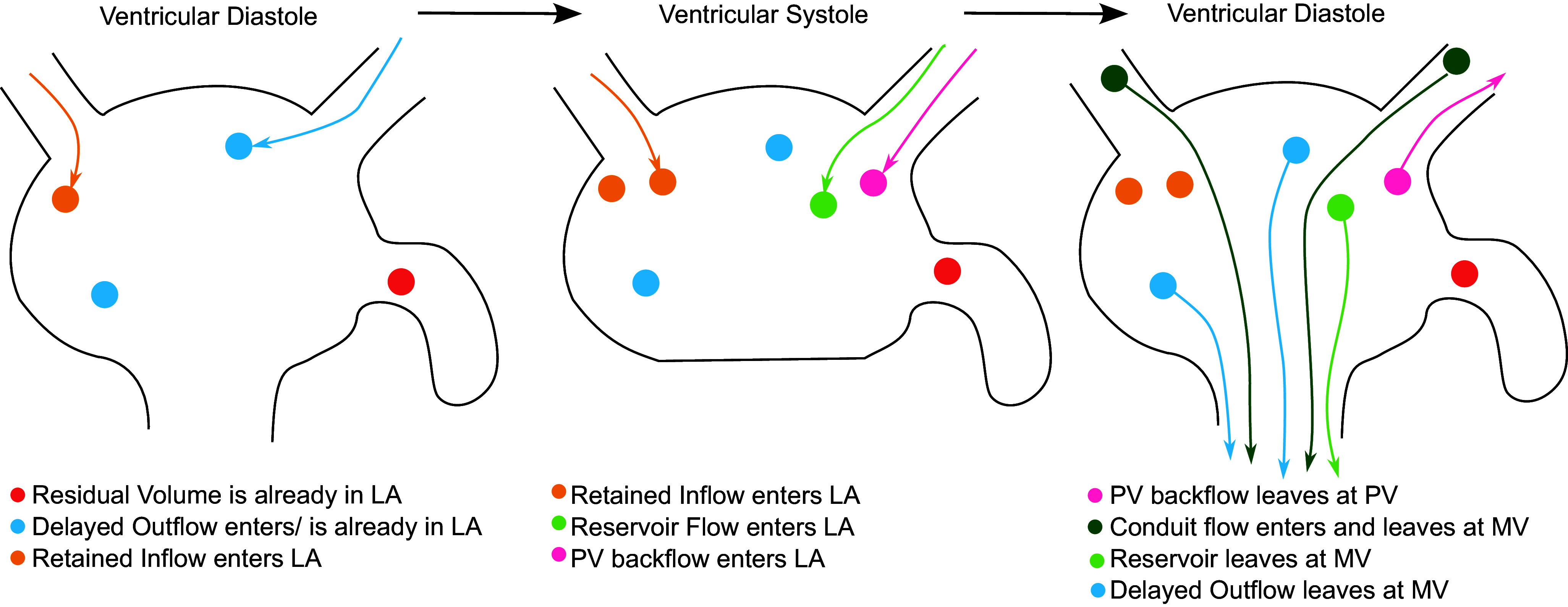
Definition of different flow components. Conduit flow enters and leaves left atrium (LA) during diastole. Reservoir flow enters LA during systole and leaves during diastole. Delayed outflow enters before systole and leaves during diastole. Retained inflow enters LA during previous cardiac cycle but does not leave. Residual volume stays in LA over 2 heartbeats. Pulmonary vein (PV) backflow leaves through pulmonary veins. MV, mitral valve.

The atrial blood volume was separated into six different components, as displayed in Fig. 1. Conduit flow was computed from the particles seeded in the pulmonary veins and defined as blood entering the LA at the pulmonary veins during ventricular diastole and leaving it through the mitral valve during the same diastolic phase. From the pulmonary vein seeding, blood entering the LA through the PV and returning to the PV during any time point in the cardiac cycle was defined as PV back flow. Based on the volume-based computed particle trajectories, blood entering the atrium during ventricular systole and leaving in the following diastole phase was defined as reservoir flow. Delayed outflow was defined as blood that leaves the atrium during diastole and either entered in the previous diastole or was already in the atrium before that. Retained inflow was blood that entered the atrium in the previous cardiac cycle but did not leave. Finally, blood volume that stayed in the atrium during the two tracked cardiac cycles was defined as residual volume. Blood from the LA that left through the pulmonary veins was added to the PV backflow component.

The results of the ventricular flow components are commonly presented as percentage of the end-diastolic ventricular volume, since this is equal to the sum of the volume of all components. Because the left atrium also experiences conduit flow, the sum of the atrial components will not be equal to the maximum atrial volume. Thus, there is no obvious factor to normalize the atrial flow components with. Possible factors of normalization are the volume of the left atrium at the end of ventricular systole, the patient’s body surface area (BSA), or the total amount of blood traveling through the atrium in one heartbeat, which is equivalent to the left ventricular stroke volume (LVSV).

For each particle, the instantaneous kinetic energy was calculated by the following equation:

(*2*) $${\text{KE}}_{\text{particle }}(t)=\frac{1}{2}\rho \times v{\left(t\right)}^{2}\times \text{V}$$

where ρ is the blood density, *v* is the velocity at a given time *t*, and V is the volume the particle represents. To compare the kinetic energy of the different components, the kinetic energy of all particles inside the LA at a time point was summed up and divided by the volume represented by these particles.

### Calculation of Volume-Based Metrices

Atrial blood flow is commonly analyzed using 2-D or 3-D echocardiography using parameters such as atrial diameter, area, and volume, as well as atrial and pulmonary flow (6). For comparison, parameters used in echocardiography that are similar to the flow components were calculated based on the time-resolved 3-dimensional CT images (see Table 1).

**Table 1.** Volumetric indices of atrial function related to flow components

Parameter	Equation	Related Atrial Function
LA EF, %	$\frac{{\text{LA}}_{\text{max}}-{\text{LA}}_{\text{min}}}{{\text{LA}}_{\text{max}}}\;\times 100$	Global function, reservoir
LA expansion index	$\frac{{\text{LA}}_{\text{max}}-{\text{LA}}_{\text{min}}}{{\text{LA}}_{\text{min}}}$	Reservoir function
LA passive EF, %	$\frac{{\text{LA}}_{\text{max}}-{\text{LA}}_{\text{pre-A}}}{{\text{LA}}_{\text{max}}}\;\times 100$	Conduit
LA active EF, %	$\frac{{\text{LA}}_{\text{pre-A}}-{\text{LA}}_{\text{min}}}{{\text{LA}}_{\text{pre-A}}}\;\times 100$	Booster pump
LV stroke volume − LA stroke volume, mL	${\text{LV}}_{\text{Stroke Volume }}-({\text{LA}}_{\text{max}}-{\text{LA}}_{\text{min}})$	Conduit

EF, ejection fraction; LA, left atrium; LV, left ventricle; LA_max_, maximum atrial volume; LA_min_, minimum atrial volume; LA_pre-A_, LA volume at diastasis.

Furthermore, the absolute passive and active atrial volume changes were calculated as LA_max_ − LA_pre-A_ and LA_pre-A_ − LA_min_, respectively. This was to better understand whether the differences between the groups in passive and active EF were due to changes in the atrial volume or atrial absolute volume.

### Statistical Analysis

The statistical analysis was conducted in MATLAB Version R2022b. The groups were compared using the two-sample *t* test with a significance level of 0.05.

## RESULTS

### Patient Characteristics

The two analyzed groups had similar patient characteristics (Table 2). However, the body mass index of the AF group was significantly higher than in the control group. In both groups, there were more men than women, and on average, the AF group was 7 yr older than the control group, but this difference was not statistically significant. The atrial volume in the AF group was higher than in the control group, whereas the atrial ejection fraction (EF) was lower. The passive atrial EF was smaller in the AF group, whereas the absolute passive volume change was similar between the groups. An inverted pattern was found for the active atrial volume change, with no difference in the active EF but a significantly higher active atrial volume change in the AF group. Furthermore, the blood residence time was higher in the AF group (17).

**Table 2.** Participant characteristics

	Controls	AF Group	*P* Value
*n*	8	21	
Age, yr	60 ± 14	67 ± 9	0.15
Women, *n* (%)	3 (38)	6 (29)	0.84
Body surface area, m^2^	2.13 ± 0.28	2.02 ± 0.17	0.22
Height, m	1.73 ± 0.07	1.78 ± 0.09	0.17
Body mass index	32 ± 6	26 ± 2	**<0.001**
Heart rate, beats/min	67 ± 7	62 ± 8	0.16
Systolic blood pressure, mmHg	139 ± 17	145 ± 20	0.47
Diastolic blood pressure, mmHg	76 ± 15	81 ± 12	0.37
LA_max_ volume (index), mL/m^2^	41 ± 7	66 ± 15	**<0.001**
LA ejection fraction, %	53 ± 7	43 ± 9	**0.007**
LA expansion index	1.18 ± 0.3	0.79 ± 0.27	**0.002**
LA passive ejection fraction, %	23 ± 9	15 ± 4	**0.002**
LA passive volume change, mL	20 ± 9	19 ± 5	0.76
LA active ejection fraction, %	39 ± 5	33 ± 8	0.07
LA active volume change, mL	26 ± 4	37 ± 9	**0.002**
LV stroke volume, mL	98 ± 26	106 ± 20	0.42
LV stroke volume − LA stroke volume, mL	53 ± 19	49 ± 14	0.60
LA residence time (cardiac cycles)	0.72 ± 0.14	1.2 ± 0.27	**<0.001**

Values are means ± SD. AF, atrial function; LA, left atrium; LV, left ventricle; LA_max_ volume, maximum atrial volume the intergroup comparisons were made using a 2-sample *t* test. *P* values < 0.05 are shown in boldface. Atrial max volume was indexed by the body surface area.

### Flow Components

**Figure 2.**
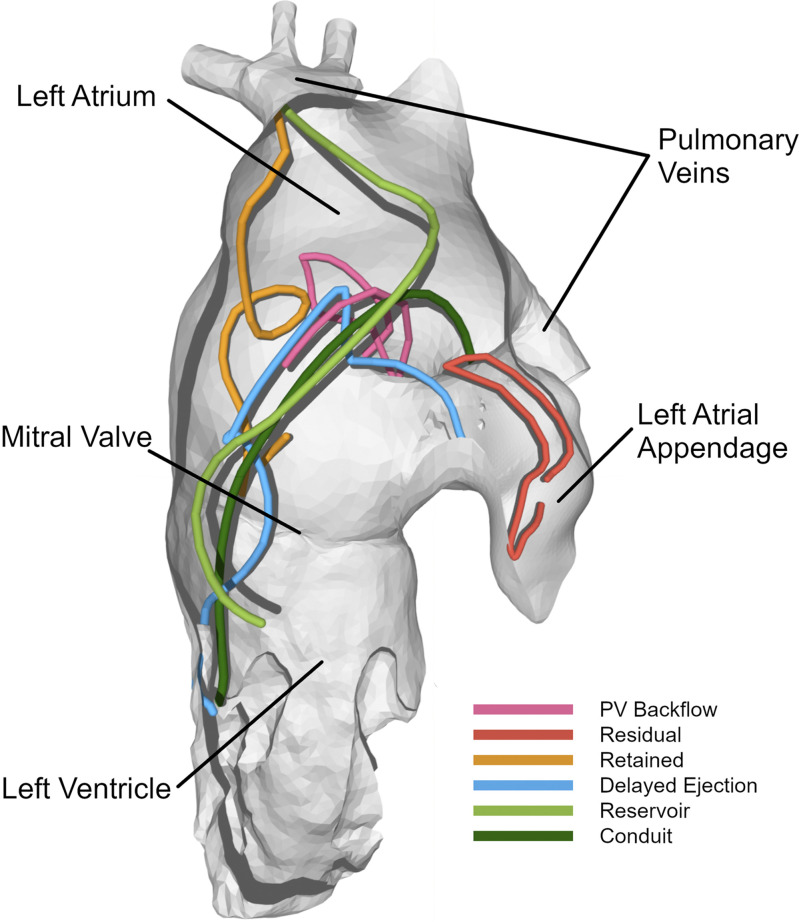
Three-dimensional (3D) volume rendering of endocardial surface segmentation and flow paths of one particle per component in a patient from the atrial fibrillation (AF) group during systole and following diastole. Surface segmentation at mid diastole from the viewpoint of the ascending aorta.

Particle traces were computed in all datasets and divided into flow components. The path traveled by one representative particle per component in one participant is shown in Fig. 2 through a 3-D visualization. For anatomical orientation, a surface segmentation of the CT image is also shown.

**Figure 3.**
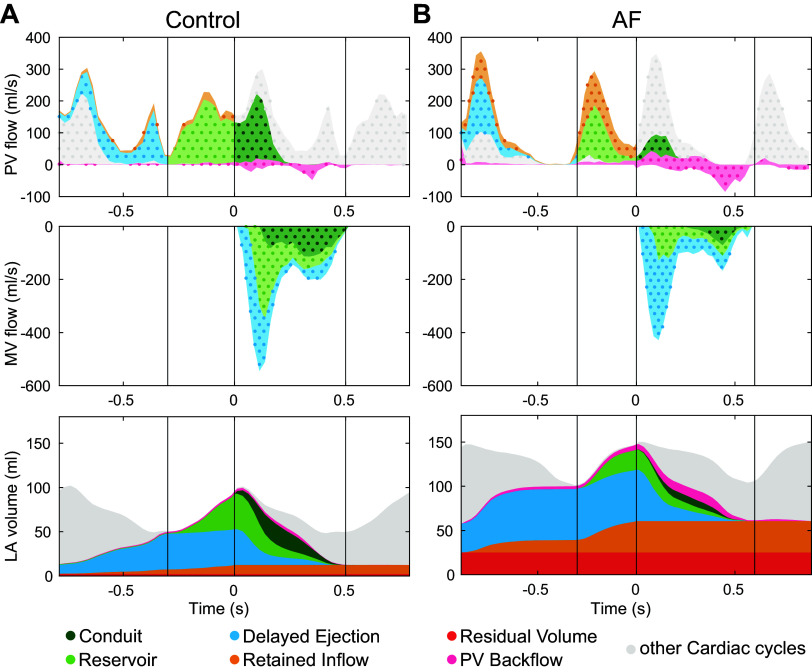
Atrial flow components over time. Temporal course of flow components in control (*A*) and patient with atrial fibrillation (AF; *B*). *Top*: component flow rates entering the left atrium (LA) at the pulmonary veins (PV). *Middle*: component flow rates leaving the LA at the mitral valve (MV). *Bottom*: component composition in the atrial volume over time. Dotted areas indicate flow rates, whereas solid color surfaces represent volumes. Systole and diastole refer to ventricular systole and ventricular diastole, respectively. Gray areas indicate flow components that are part of the previous or following cardiac cycle.

To understand when flow components entered and left the atrium, Fig. 3 illustrates the flow rates at the PV and MV of the flow components and how the atrial volume is composed of the different components over time. To cover both the inflow and the outflow, two cardiac cycles were displayed. The blood components left the LA in the order that they entered it, with the early diastolic flow being mainly composed of delayed ejection and reservoir flow, whereas the late diastolic flow was dominated by reservoir and conduit flow. The retained inflow component and residual volume were larger in patients with AF than in the control subjects. For both participants, some blood flew back into the pulmonary veins toward the lungs, mainly during late diastole. Most of this blood entered during early diastole; only a small amount entered during the previous cardiac cycle.

**Figure 4.**
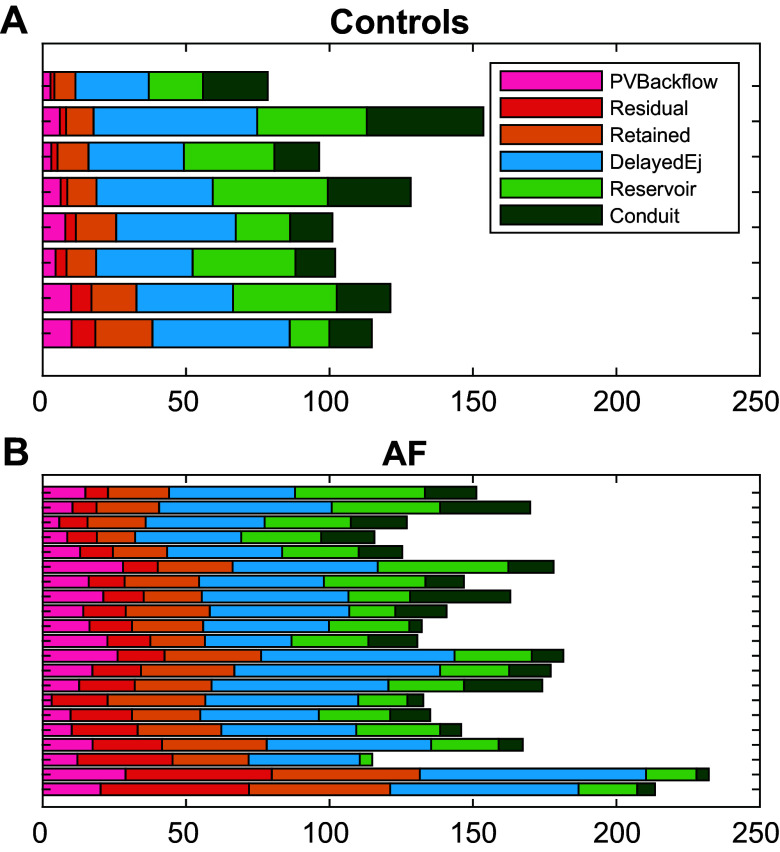
Volume of flow components for each participant of the control group (*A*) and group of patients with atrial fibrillation (AF; *B*).

**Figure 5.**
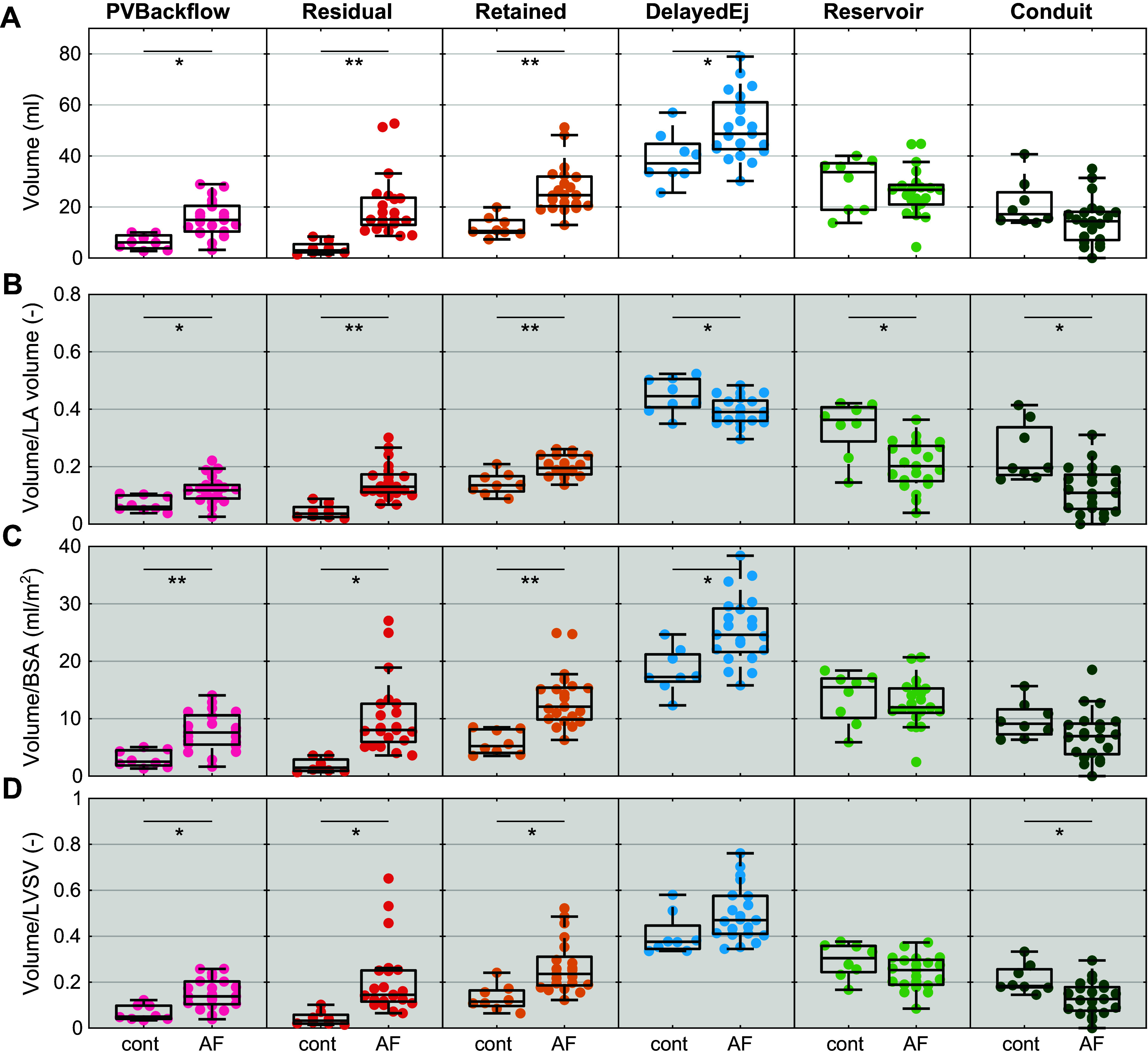
Statistical comparison of flow components between controls and patients with atrial fibrillation (AF). *A*: absolute volume, which was used in the further analysis. *B*: normalized by left atrium (LA) volume at end-ventricular systole. *C*: normalized by body surface area (BSA). *D*: normalized by left ventricular stroke volume (LVSV). *t* Test comparison of component volume between the 2 groups. *P < 0.05; **P < 0.001. Specific *P* values are displayed in Table A1. PV, pulmonary veins.

For the control participants, the volumes of conduit, reservoir, and delayed ejection were larger than the volumes of PV backflow and residual and retained inflow (Fig. 4*A*). In the AF group, the sum of the components was larger, mainly due to larger volumes in PV backflow and residual and retained inflow (Figs. 4*B* and 5*A*). The absolute volume of the reservoir and conduit flow was similar between the groups. After normalizing the atrial flow component by the atrial volume at end-ventricular diastole, all components showed a significant difference between the groups (Fig. 5*B*). Comparing the volume normalized by BSA to the absolute values, the general pattern was similar, with significantly larger volumes of PV backflow, residual volume, retained inflow, and delayed ejection in the AF group (Fig. 5*C*). When normalizing by left ventricular stroke volume, the conduit flow was significantly smaller in the AF group (Fig. 5*D*).

### Kinetic Energy

**Figure 6.**
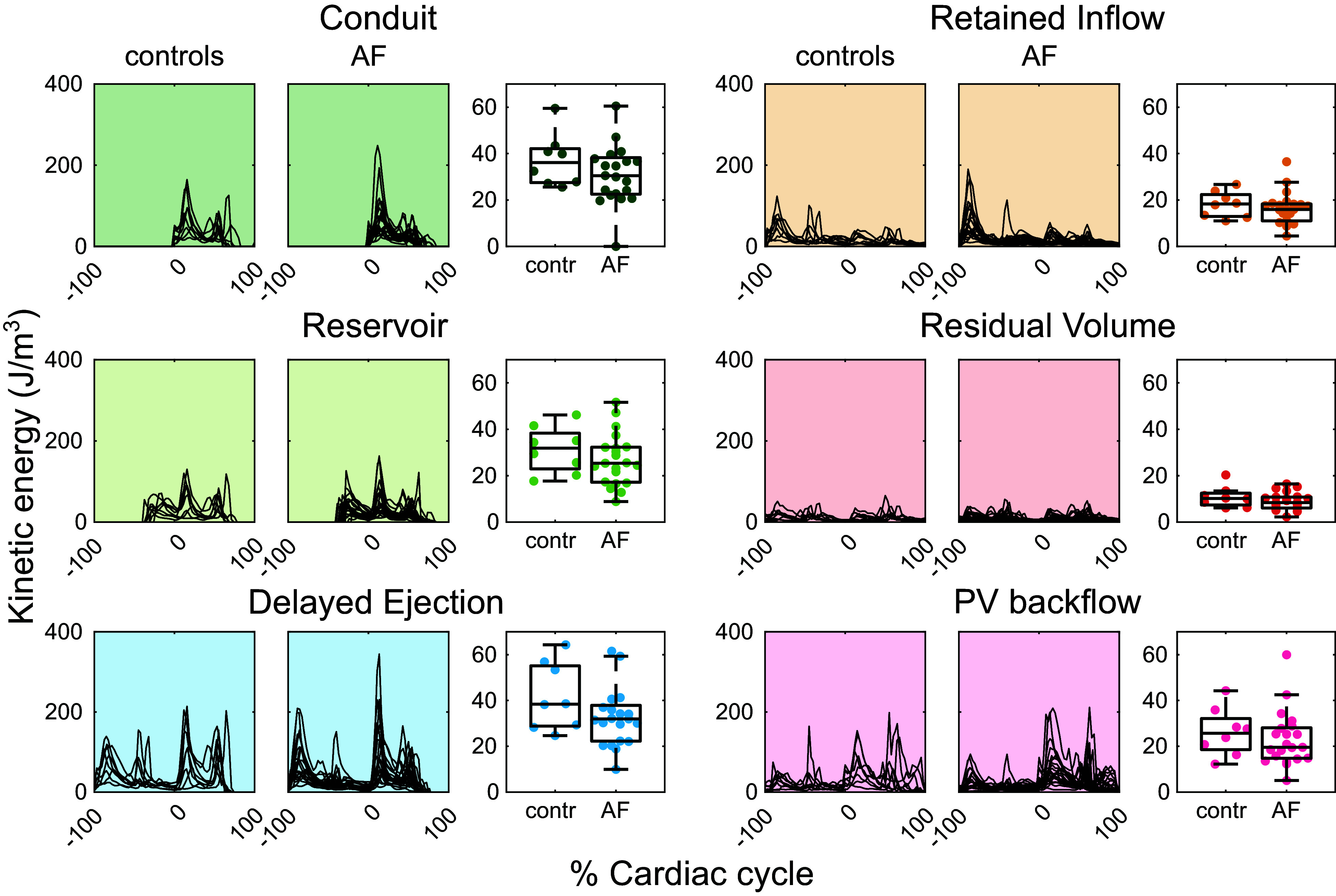
Kinetic energy (KE) over time for each component over 2 consecutive cardiac cycles normalized by the volume of the component and boxplot comparison of time-average KE between the 2 groups. Each black line refers to 1 participant, and the length of the cardiac cycle was normalized to 100%. In the previous cardiac cycle, components such as reservoir and retained inflow enter the left atrium (LA). In the following cardiac cycle, conduit, reservoir, and delayed ejection leave the LA. The time-average KE was calculated by averaging the KE at the times when the component was inside the LA. There was no significant difference between the time-average KE of each component between the groups.

For all components, the kinetic energy per volume was similar between the groups, as can be seen in the similar time-average kinetic energy distributions, but it differed between the different components (Fig. 6). Conduit and delayed ejection had the highest kinetic energy. For most participants, reservoir and delayed ejection peaked both during early and late peak ventricular filling, with lower kinetic energy during diastasis. The residual volume had overall very low kinetic energy.

### Comparison with Volumetric Indices

**Figure 7.**
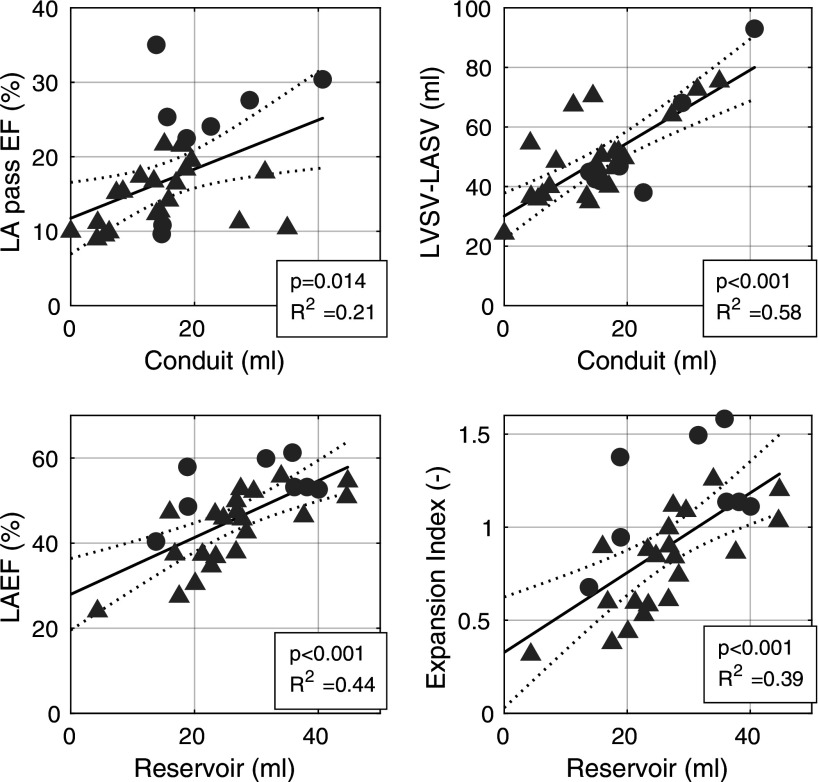
Linear regression of flow components conduit and reservoir with volumetric indices of atrial function that are associated with these atrial functions, derived from CT-based volumes. EF, ejection fraction; LVSV, left ventricular stroke volume; LASV, left atrial stroke volume (difference of minimum and maximum atrial volume); LA, left atrium; AF, atrial fibrillation.

Atrial conduit function has been associated with LA passive ejection fraction and the difference between left ventricular stroke volume and left atrial volume change, whereas atrial reservoir function has been associated with LA ejection fraction and expansion index. To compare the volume of conduit and reservoir flow, derived from flow component analysis, with these parameters, linear regression was performed, as shown in Fig. 7. There were significant associations between all compared parameters, with varying strength in the coefficient of determination. The strongest association was found between conduit volume and LVSV − LASV, with an *R*^2^ of 0.58.

## DISCUSSION

Atrial blood flow is an important part of cardiac physiology but has previously not been studied in depth using blood particle trajectories. In this study, we seeded particles in the atrial volume and from the pulmonary veins to identify six atrial blood components, including conduit and reservoir flow, providing new insights into the physiological mechanisms of atrial blood flow in patients with and without atrial fibrillation.

Previously, two studies have analyzed some flow components in the LA. Dillon-Murphy et al. (15) investigated two patients with AF, whereas Gaeta et al. (16) investigated three young healthy volunteers. Both studies presented their results as percentages. For the comparison with our results, we calculated the absolute volumes of these components, which can be found in the Appendix (Tables A2 and A3). The method from Dillon-Murphy et al. allows for comparison of the volume-based flow components. They had similar volumes of delayed ejection and reservoir flow (they refer to it as direct flow), as we found in our study. The residual volume of *patient A* was similar to our control group, whereas that of *patient B* was similar to the AF group. The retained inflow of *patient A* was similar to our results, whereas the value for *patient B* was larger than of any participant in our study. This could be because of the very large atrial volume (197 mL) of this participant. The direct flow in the study by Gaeta et al. is the same as the combination of reservoir and conduit flow in our study, and the values are similar to the ones in our study. The PV backflow was smaller than in our study, which could be explained by the much younger participant ages in the study of Gaeta et al. In general, the volumes found in our study are comparable with what has been published before.

Furthermore, the relation of conduit and reservoir flow to atrial functional indices used in echocardiography was examined. Conduit flow was shown to be associated with LA passive EF and LVSV − LASV. The association to LVSV − LASV was stronger, which is reasonable because this parameter is considered the most correct volume-based estimate of conduit flow. The reservoir volume was similarly strongly associated with both LAEF and LA expansion index, which is reasonable as these two parameters are relatively similar. The LA passive EF and the LA expansion index differed between the controls and AF group, whereas the absolute volumes of conduit and reservoir flow did not differ. The large difference in LA passive EF seems to be driven by a large difference in atrial volume, as the absolute passive volume change is not significantly different while the atrial volume is different (Table 2). When normalizing conduit and reservoir flow with the atrial volume (Fig. 5*B*), there also appears a significant difference between the groups. Thus, the calculation of conduit and reservoir flow matches well with the volume-based parameters used in echocardiography to estimate these quantities.

The conduit and reservoir flow are the components with the most direct flow, thus least linked to increased stasis. In agreement with the traditional definition of conduit flow, most of it enters the left atrium in early diastole. With our method, we could show that this blood volume then leaves the atrium in late diastole. The reservoir flow enters while the mitral valve is closed and leaves when it is open. In the control group, the average volume of this component is twice as large as the retained inflow, indicating that most blood that enters during systole is actual reservoir flow. Although the volume of the LA was larger in the AF group, absolute volumes of conduit and reservoir flow were similar between the groups. When normalizing with LVSV, an indicator for the amount of blood passing through the LA, conduit volume appeared to be slightly smaller. In general, it seems like these components are not very strongly affected by the increased volume.

Delayed ejection, retained inflow, and residual volume all spent at least one cardiac cycle inside the LA and the volume of these components was increased in the AF group. Retained inflow and residual volume had lower kinetic energy than other components, potentially increasing the risk for stasis. Although the volume of the components differed between the groups, the relative kinetic energy was similar between the groups for all components. This indicates that although the volumes of the components are altered, the energetic characteristics are similar between the groups. Our results suggest that the increased atrial volume predominantly increases the volume of stagnant components, resulting in an increased residence time in these patients.

At late diastole, some blood tends to flow back toward the pulmonary veins. The amount of PV backflow was larger in the AF group. Maruyama et al. (18) found that increased PV backflow could predict transition from paroxysmal to permanent AF in patients with hypertension, highlighting the PV backflow as an interesting biomarker in paroxysmal AF . As shown in Table 2, the atrial volume change during early diastole (ventricular relaxation) was similar between the groups, whereas the patients with AF had a larger late diastolic volume change (atrial contraction). The PV backflow occurred in the late diastolic filling phase, indicating that in this group, the atrium contracts more than normal, pushing blood toward the pulmonary veins instead of only to the ventricle. This effect could be worsened by potential ventricular relaxation abnormalities, where the atrial contribution to ventricular filling is larger relative to the LV relaxation.

The flow components of the left ventricle are commonly presented as percentage of the end-diastolic ventricular volume. Applying the same principle to the left atrium poses an issue. In patients with atrial fibrillation or atrial cardiomyopathy, the atrium is commonly enlarged, a pattern that is also observed in the current study (Table 2). Figure 5*B* shows the flow components normalized by the LA volume at end-ventricular systole. After this normalization, the volumes of conduit and reservoir flow are significantly smaller in the AF group, whereas they were similar to controls when not normalized. This indicated that these differences are caused more by the difference in atrial volume rather than the volume of the components. As the atrial volume varies between individuals, for example, depending on the individual’s body size, it might be beneficial to normalize the volume of the components to be able to make an individual risk assessment. One possible factor of normalization could be the body surface area, as shown in Fig. 5*C*. Alternatively, the components can also be normalized by the left ventricular stroke volume, since this is the amount of blood that travels through the left atrium during one cardiac cycle, excluding significant mitral regurgitation and shunts (Fig. 5*D*). Studies in a larger population and with a clinical outcome as an end point are needed to identify which metric of normalization is most appropriate. Anyhow, in the current study, we could show that significant differences between patients with AF and control subjects are observable when analyzing the absolute volume of the components as well as using normalization.

There are some limitations in our current study. First, the flow component model does not take mitral regurgitation into account since this was an exclusion criterion for participation. However, adding regurgitant flow to the model is fully possible. Furthermore, our study has a relatively small number of participants and there is no follow-up data on patient outcomes. However, the population size was sufficient to develop and evaluate the flow component model and demonstrated significant differences between patients with AF and control subjects. In addition, the control group had an indication for a clinical CT, indicating that the participants might have some other cardiac diseases and are not fully healthy. This might lead to some differences between the groups other than caused by AF. Although comparing with a fully healthy group would be optimal, finding age-matched healthy controls is challenging because of the high prevalence of cardiovascular diseases in this age group. Tracking atrial blood flow for a full cardiac cycle forward and backward in time allowed for the analysis of the amount of blood traveling through the LA from the pulmonary veins to the mitral valve, which was not possible in previously published studies (15, 16). The bias between the inflow and outflow volume, occurring because of numerical inaccuracies, was relatively low when compared with the size of the total flow volume and the volume of the different components. The standard deviation of the difference between inflow and outflow in the AF group was, however, larger than in the control group. This might be related to the small group sizes, but the inaccuracy could also be dominated by inaccuracies in the computation of the components that differ between the groups. Analysis of different temporal resolutions showed a decrease in difference between inflow and outflow. In future studies, it could be beneficial to compute path lines with a temporal resolution of 0.01 s, although this might increase the computational time. Since the difference in inflow and outflow is smaller than most flow components, the method is still accurate enough to analyze the blood flow in the left atrium.

In conclusion, left atrial flow component analysis reveals new information on atrial function and dysfunction. Using this methodology, it was shown that conduit flow, blood that enters and leaves the left atrium within the same diastolic phase, exists. Compared with controls, for patients with paroxysmal AF in sinus rhythm, the conduit flow volume was not affected, whereas the residual volume and blood volume flowing back toward the pulmonary veins were significantly larger. Atrial flow component analysis allows for efficient comprehensive assessment of the complex 3-D and time-varying atrial flow fields, thus enabling in-depth exploration into the role of atrial blood flow in atrial function, disease progression, and stroke risk with the ultimate goal of patient-specific risk stratification and disease management, such as anticoagulation treatment, in atrial fibrillation and other atrial diseases.

## DATA AVAILABILITY

Data will be made available upon reasonable request.

## GRANTS

This research was funded by Region Östergötland Grants 974839 and RÖ-987498, Swedish Heart and Lung Foundation Grants 20200220 and 20210441, Swedish Research Council Grants 2018-02779 and 2022-03931, and Sweden’s Innovation Agency Vinnova Grant 2019-02261.

## DISCLOSURES

No conflicts of interest, financial or otherwise, are declared by the authors.

## AUTHOR CONTRIBUTIONS

S.B., J.L., and T.E. conceived and designed research; S.B., I.S., L.H., L.O.K., and C.-J.C. performed experiments; S.B., J.L., C.-J.C., and T.E. analyzed data; S.B., J.L., C.-J.C., and T.E. interpreted results of experiments; S.B. prepared figures; S.B. drafted manuscript; S.B., J.L., I.S., L.H., A.P., L.O.K., C.-J.C., and T.E. edited and revised manuscript; S.B., J.L., I.S., L.H., A.P., L.O.K., C.-J.C., and T.E. approved final version of manuscript.

## Figures and Tables

**Figure A1.**
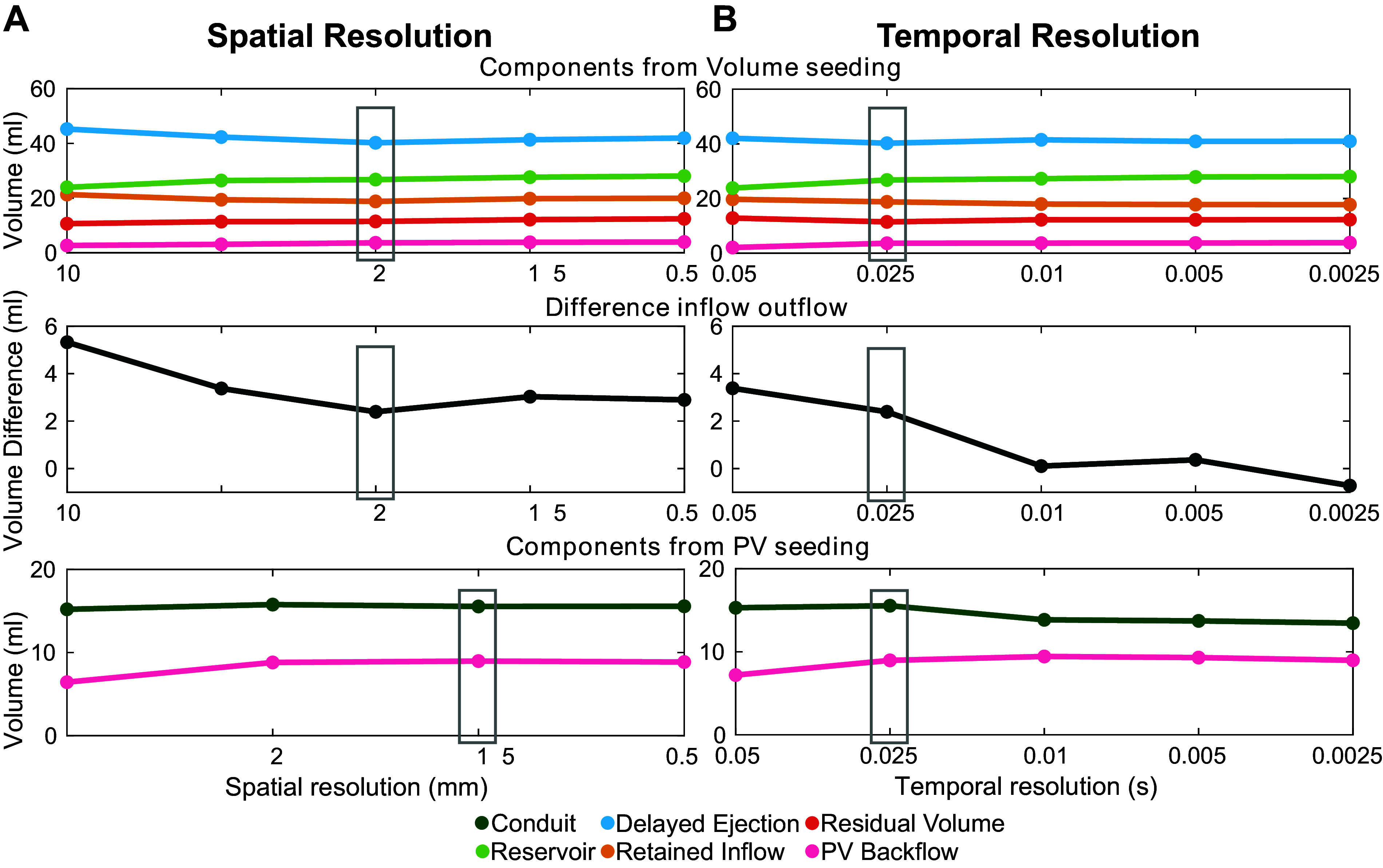
Volume of flow components and difference between inflow and outflow for different spatial resolutions at temporal resolution 0.025 s (*A*) and different temporal resolutions at spatial resolution 2 mm (*B*). The resolutions of the final calculations are marked by the gray boxes.

**Figure A2.**
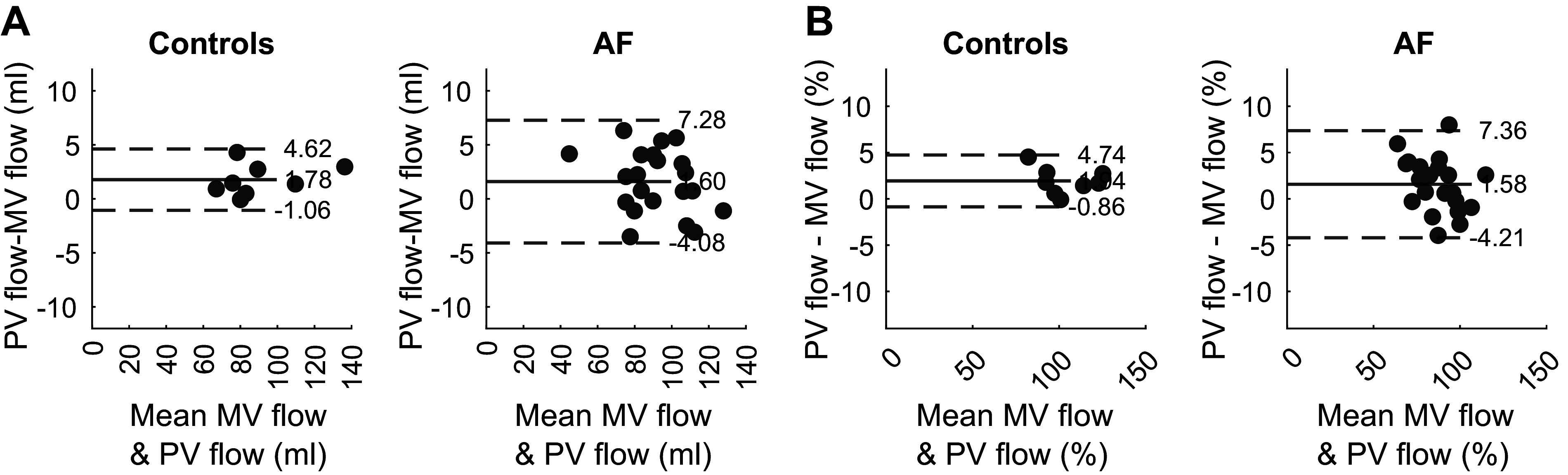
Bland–Altman plot comparing the particle trace-based amount of blood flowing in at the pulmonary veins (PVs) and leaving at the mitral valve (MV; *A*) and comparing the flow volumes in relation to the maximal atrial volume (*B*). AF, atrial fibrillation.

## APPENDIX

### Code Verification

To verify the accuracy of the flow component analysis, the volume of the flow components and the difference between atrial inflow and outflow were calculated (Fig. A1). For the pulmonary veins, it was not possible to calculate the flow components at a resolution of 10 mm since this led to some pulmonary veins having very few pathlines.

To control the quality of the results, we computed the total blood volume passing through the pulmonary veins and mitral valve in a cardiac cycle. In agreement with the conservation of mass, these two values should be the same. As shown in Fig. A2, on average, the flow through the pulmonary veins was larger than that through the mitral valve, with a bias of 1.78 mL for the control group and 1.52 mL for the AF group. In relation to the atrial volume, the bias was 1.94% for the control group and 1.58% for the AF group, with a spread of 2.8% and 5.78%, respectively. However, in some individuals, the flow through the mitral valve was larger than at the pulmonary veins. In the AF group, the standard deviation of the difference between the two flows is larger than that in the control group.

### Normalized Volume of Flow Components

Figure 5 displays the statistical comparison of the flow components between control subjects and patients with AF, normalized by various factors. Table A1 contains the corresponding *P* values of the different comparisons.

**Table A1.** P values of comparison of volume of flow components between groups normalized by various factors

	PV Backflow	Residual	Retained	Delayed Ejection	Reservoir	Conduit
Absolute volume	**1.0e-03**	**8.9e-04**	**2.3e-04**	**0.019**	0.424	0.085
Volume/LA volume	**0.011**	**7.9e-05**	**3.5e-04**	**0.016**	**1.3e-03**	**1.3e-03**
Volume/BSA	**6.5e-04**	**1.1e-03**	**2.3e-04**	**0.003**	0.568	0.121
Volume/LVSV	**0.002**	**0.006**	**0.003**	0.059	0.093	**0.008**

PV, pulmonary veins; LA, left atrium; BSA, body surface area; LVSV, left ventricular stroke volume. Boldface indicates significant values (*P* < 0.05).

### Comparison with Other Studies

Two other studies have calculated some flow components in the left atrium before. Dillon-Murphy et al. (15) computed the components based on the atrial volume (Table A2).

**Table A2.** Comparison to results by Dillon-Murphy et al.

	*Patient A*		*Patient B*		Our Study	
	%	mL	%	mL	Controls, mL	AF, mL
LA volume		113		197	103 ± 22	158 ± 35
Delayed ejection	35.3	39.9	26.6	52.4	39 ± 10	52 ± 13
Residual volume	7.7	8.7	16.4	32.3	4 ± 3	20 ± 12
Reservoir (direct flow)	15.3	17.3	18.6	36.6	29 ± 10	26 ± 9
Retained inflow	35.6	40.3	38.4	75.6	12 ± 4	27 ± 9

AF, atrial fibrillation; LA, left atrium.

Gaeta et al. (16) tracked particles from the pulmonary veins, leading to three different flow components (Table A3).

**Table A3.** Comparison to results by Gaeta et al.

	Subject			Our Results	
	1	2	3	Controls, mL	AF, mL
Reservoir + conduit, mL	45.2	43.4	37.1	50 ± 17	40 ± 15
PV backflow, mL	2.95	2.5	0.4	6 ± 3	16 ± 7

AF, atrial fibrillation; PV, pulmonary veins.

It was not possible to compare our results with the retained inflow computed by Gaeta et al. since they started the calculation at the beginning of systole, whereas we started at the beginning of diastole.
